# A Medical Causerie

**Published:** 1915-05-01

**Authors:** 


### A MEDICAL CAUSERIE.

There is trouble on hand in the world created by the National Insurance Act, and this time, we are assured, the panel doctors really mean business. The most acute of the controversies, here as in so many other directions, arises out of the war. This has carried many thousands of men beyond the jurisdiction of the Insurance Commissioners, and has in corresponding measure relieved the panel doctors. It is therefore reasonable enough that the doctors concerned, having their work diminished, should have also their remuneration diminished. On this point the Insurance Commissioners and the doctors are agreed. But what the Commissioners propose to do is to make an arbitrary reduction from 1s. 9d. per head for the quarter to 1s., and this reduction is to apply in every instance whatever the character of the practitioner's list; whether 50 per cent. or 5 per cent. of his patients have gone to the war, he is to be reduced to 1s. per head. As opposed to this the doctors say they are quite willing to suffer a reduction of payment provided this is in proportion to the diminution of their lists as a consequence of the Army claims. Under the Commissioners' scheme a man whose insurance responsibilities are not affected by the war will lose three-sevenths of his insurance income. Last week a vigorous meeting of those concerned was held, and the note of defiance was high. Whether there will be equal vigour in action remains to be seen. Those who fight and run away may, it is true, live for a second occasion, but no one will confess confidence in them as trustworthy and redoubtable warriors. Apart from this particular disturbance there are evidences of the most serious muddle in the central Insurance Offices. Indeed, individual practitioners are often quite unable to know who officially is, and who is not, on their panel, and are consequently not in a position to determine whether their quarterly cheques are accurate or inaccurate. Red tape has its advantages, but when it is all frayed and tangled the position is worse than ever. A still further evidence of the existing tension is the resignation of the Chairman of the London Insurance Committee, who finds himself the subject of what he at least regards as a vote of censure. In the London area there is a deficit in the Drug Fund to the tune of £53,000, and though a committee has been appointed to deal with this situation, it has no means of raising funds. The chemists charge the doctors with over-prescribing; the doctors deny this, and little attempt has been made to substantiate the suggestion. Further, in the early days when visions of first-class hotels and free choice of doctors were bright points on the horizon, the promise of the best drugs, too, took its due place. It would be sad now openly to fall back on a second-class article.

The proposal to equip in London further hospitals for sick and wounded soldiers and to staff these with consulting physicians and surgeons who are not officers in the Army or in the Territorial Forces is still under consideration, but it has hardly yet taken practical shape. The great advantage of such an arrangement would be that the physicians and surgeons would be free, as at present, to continue their responsibilities in the civil hospitals. In short, they would do exactly the same work as the present staff of the military general hospitals, but they would not wear uniforms, would not hold military rank, and would not, presumably, draw military pay. The proposal seems an excellent one, for it secures attention to the soldiers without prejudicing still further the clinical services of the non-military hospitals. Some of the juniors, it is said, are disappointed with the absence of rank, but the great majority of those interested see clearly that they can give what is needed by the present emergency, and can at the same time continue their ordinary and not less necessary duties. The War Office has at last come to recognise that in part-time service is the solution of many of its difficulties, at least on the medical side; the hard and self-satisfied tone of a few months ago has necessarily been modified, but there is room for some improvement even yet. Many practitioners who are now offering their services are doing so at considerable personal loss and inconvenience, and in response to an appeal issued officially by the Director-General of the R.A.M.C. Subordinates might get a hint to bear this in mind. Men who from patriotic motives respond to an invitation for help are not to be addressed and treated as though they were derelicts seeking for a job.

Among the various controversies which have arisen in connection with the treatment of wounds little has been heard of the value of ionisation in promoting the closing of sinuses and similar rebellious lesions. Yet those who have worked with this method speak of it in the most enthusiastic terms. Some of the new military officers are well equipped in this direction, and as patients are not wanting there is every reason to anticipate that a definite conclusion, one way or the other, will be reached. One of the debts the profession owes to the late Dr. Lewis Jones is the introduction of ionisation, and indeed generally it is to him that electrical therapeutic methods have been raised to the position which they occupy to-day. Only too often in the past they have been the prey and sport of the ignorant and impudent quacksalver.

The medical future of the war in relation to the home population is giving the authorities some anxiety. Epidemic disease in the North of France and in Belgium will, if it breaks out, be much more difficult to deal with now or in the future than in August last. Exposure, hardships, irregular feeding are conditions promoting rapid extension, and the Channel offers no effective barrier. Someone has proposed compulsory anti-typhoid inoculation for the whole population, but he has modestly kept his name in the background. Only in this way has he escaped the anathemas of certain well-known makers of verbal thunderbolts.

